# New products

**DOI:** 10.1155/S1463924695000216

**Published:** 1995


Journal of Automatic Chemistry, Vol. 17, No. 3 (May–June 1995), pp. 119–122

### New products

### P.S. Analytical Ltd and Inchcape Testing Services

Since its formation in 1983, P.S. Analytical has been co-operating with its customers to solve complex analytical problems. One of its initial clients was Inchcape Testing Services in St. Helens who were one of the first companies to purchase the PSA Hydride Vapour Generator. This product and its successors have been in continual use at Inchcape Testing Services.

In response to the increasing demand for lower and lower detection limits for analytes such as mercury, selenium, antimony and arsenic in environmental samples, especially groundwaters, Inchcape has sought to enhance its capability in this area. Staff at St. Helens spent many hours evaluating alternative methods for this work. After these tests and co-operation with P.S. Analytical, Inchcape agreed to purchase the combined Merlin Excalibur instrument. This allows a highly flexible construction with extensive capabilities to analyse the various elements in difficult matrices and with a high degree of accuracy and precision, even at sub ppb levels.

Inchcape Testing Services has the specific aim of being the most skilled and innovative team of professionals in environmental analytical measurement. Purchasing the P.S. Analytical Merlin Excalibur instrumentation not only provides quality instrumentation for the job but also adds to the considerable resources of P.S. Analytical's applications expertise.

*Literature on the P.S. Analytical product range is available from P.S. Analytical, Arthur House, Unit 2.03 Crayfields Industrial Park, Main Road, St Pauls Cray, Orpington, Kent BR5 3HP, UK. Tel.: 01689 891211; fax: 01689 896009.*

*Information about Inchcape Testing Services is available from Ken Hepburn, Technical Manager, Inchcape Testing Services Environmental Laboratories, Lancots Lane, St. Helens, Merseyside WA9 3ES, UK.*

### Atomic-emission detector

Hewlett-Packard has published an index of HP 5921A atomic-emission detector (AED) application notes and scientific articles. The index is a resource for chemists to obtain information on how the HP 5921A AED can be used to solve analytical challenges; it contains resource information on subject areas that include the following:

(1) Sulphur, oxygen, lead, nickel, vanadium, iron and boron in the petroleum industry.

(2) Process impurities in the petrochemical and chemical industries.

(3) Hydrocarbons, pesticides, organometallics and hazardous waste in air, water and soil environmental samples.

(4) Characterization of unknown compounds in foods, flavours and fragrances.

(5) Stable isotope labels in metabolite studies.

(6) Simplified quantitation using compound-independent calibration.

*The index is available free of charge (quote 5963-6601E) from PMG Logistics, Attn. Petra Butter, P.O. Box 533, 2130 AM Hoofddorp, The Netherlands.*

### Analysis of VOCs in water

Perkin-Elmer has published a new application note which discusses the *Japanese Standard Headspace Method for Analysis of VOCs in Water*. It summarizes and explains the simple and highly productive static headspace GC/MS procedure included in new regulations covering water quality testing. It includes details of the instrumentation and analytical conditions, a method summary listing target VOCs, and chromatograms and results tables illustrating replicate analyses. The advantages of headspace over purge-and-trap systems for environmental water analysis are also discussed.

*For further information, contact Perkin-Elmer Limited, Post Office Lane, Beaconsfield, Bucks HP9 1QA, UK. Tel.: 01494 679269: fax: 01494 679332.*

### Clenbuterol screening

Fisons Instruments VG Organic have released new information on clenbuterol screening in a new application note: *An Improved Method For Clenbuterol Screening Using High Resolution Selected Ion Recording*. Methodology which extends the use of GC-MS by improving the specificity of the technique using the high resolution capabilities of a magnetic sector mass spectrometer is described. Used as an anti-asthmatic and tocolytic drug in the treatment of both humans and animals, clenbuterol is a stimulant of the nervous system and a $\beta_2$-agonist. Together with other $\beta$-agonists, it also exhibits growth stimulatory effects, leading to enhanced muscle and decreased fat deposition. Consequently, clenbuterol is used to stimulate growth in farm animals, and by athletes seeking enhanced performance from the protein anabolic response to the drug. Such abuse within sport is attracting the attention of various sporting authorities, and thus requires precise investigation and monitoring.

Dosage is typically in the μg/kg range of human body weight—therefore a sensitive but specific method is needed to check for the presence of clenbuterol. The procedure described in the application note is sufficiently specific to be used for the analysis of heavily contaminated urine samples which have undergone enzymatic hydrolysis.

*For more information contact IAS, Queens Avenue, Macclesfield, Cheshire SK10 2BN, UK. Tel.: 01625 434343; fax: 01625 434335.*

### Pharmacokinetic study using HPLC/MS/MS

Fisons Instruments VG Organic's *Quantitative HPLC/ MS/MS In Support Of A Pharmacokinetic Study* has recently been announced. Mass spectrometry has long been employed as a highly sensitive and specific tool for pharmaceutical studies, and its use in this field has been well documented. The greater prominence of LC/MS/MS is rapidly becoming evident from drug discovery through metabolism and toxicological studies to drug manufacture. An explanation for this increased application is the introduction of the atmospheric pressure ionization techniques, electrospray (ES) and atmospheric pressure chemical ionization (APcl), in addition to improvements in computer technology. Electrospray is an extremely powerful method used in the analysis of thermally labile compounds due to the relatively mild ionizing nature of this interface. This is specifically important in the areas of drug metabolism and pharmacokinetics, where commonly occurring metabolites such as glucuronides and sulphates are encountered.

Formerly, ionization techniques, such as thermospray and plasmaspray, caused these conjugated metabolites to frequently revert to their aglycones, losing all their molecular mass information. Electrospray is more sensitive than fast atom bombardment and thermospray due to the greatly decreased level of background ions and its higher ionizing efficiency. Besides their high sensitivity and specificity, the atmospheric pressure ionization techniques are also so reproducible and robust that they lend themselves readily to automation. These highly beneficial features are described in Fison's new literature, in a study which uses HPLC/MS/MS in support of drug discovery.

*For more information contact IAS, Queens Avenue, Macclesfield, Cheshire SK10 2BN, UK. Tel.: 01625 434343; fax: 01625 434335.*

### SEM developments

Oxford Instruments Scientific Research Division produces large movement, custom produced stages for the SEM (Scanning Electron Microscope). This is particularly important, for example, for the semiconductor industry, where stages can be produced that hold wafers with diameters up to 12 inches, for techniques such as DRT, probing and EBIC. Stages can also be built for other applications, including electron beam lithography and the examination of large and heavy samples, such as engineering components. If the SEM chamber is too small, replacement chambers can also be supplied.

Detailed specifications are, naturally, dependant on the individual design but typically will offer sub-micron precision. Technologies available include microstepping motor drive with software controlled and co-ordinate storage, piezo electric movements, linear encoders, a laser interferometer system and microprobing.

In addition, the company also produces a wide range of standard and custom stages for the SEM and TEM, which include heating cooling, and tensile facilities.

All Oxford Instruments customers now have free and unlimited access to the company's specialist engineering and applications support hotline.

*For further information contact Oxford Instruments Scientific Research Division, Old Station Way, Eynsham, Witney, Oxon OX8 1FL. Tel.: 01865 882855; fax: 01865 881944.*

### Net Communication

The benefits of the Internet have now been realized for analytical chemists with the creation of LabNET, a dedicated global platform providing a plethora of facts on the latest product and technological developments.

LabNET goes on-line in May 1995. It is an unique interactive response mechanism giving access to manufacturers throughout the world. Clear step-by-step instructions guide visitors quickly through to their selected areas where exciting graphical and textual data may be analysed, down loaded, printed or re-visited as often as required. Direct links to companies of your choice are also available, enabling you to discuss enquiries via e-mail for the cost of a telephone call.

LabNET information is available on the 01799 516610 or e-mail ak45@solo.pipex.com.

### Thermal analysis applications

Three new thermal analysis application notes are available from Perkin-Elmer.

*PETAN 54—Epoxy Cure Characterization using Simultaneous Dynamic Mechanical Analysis-Dielectric Analysis (DMA-DEA)* describes recent developments in polymer composite manufacturing which have resulted in higher quality and lower cost epoxy composites. The method and instrumentation used to simultaneously characterize the curing process of epoxy using the Perkin-Elmer DMA 7e Dynamic Mechanical Analyzer with the cup and plate measuring system and the Micromet Instruments Eumetric System 111 Microdielectrometer are outlined.

*PETAN 55—Prepreg Cure Characterization using Simultaneous Dynamic Mechanical Analysis-Dielectric Analysis (DMA-DEA)* looks at the effects of batch-to-batch material variability and changes in the curing process on final quality of parts made from thermosetting prepregs. The note outlines the simultaneous characterization of the mechanical and dielectric curing process of an EPON 828 prepreg using the Perkin-Elmer DMA 7e Dynamic Mechanical Analyzer with the parallel plate measuring system and the Micromet Instruments Eumetric System 111 Microdielectrometer.

*PETAN 56—Determination of the Curing Kinetics of an Epoxy Adhesive using Isothermal DSC Kinetics Software* outlines the use of DSC isothermal software for the 7 Series/UNIX system to obtain the curing parameters for an epoxy adhesive.

*For further information, contact Perkin-Elmer Limited, Post Office Lane, Beaconsfield, Bucks HP9 1QA, UK. Tel.: 01494 679269; fax: 01494 679332.*

### Electrospray LC/MS analysis

An application note from Fisons Instruments VG Organic of Altrincham, Cheshire, describes the development of a selective and sensitive method for detecting glycopyrrolate, a quaternary ammonium anticholinergic drug banned in horseracing due to its respiratory enhancing properties. *Electrospray LC/MS Analysis of Glycopyrrolate in Equine Sport* describes a rapid and reliable technique appropriate for glycopyrrolate quantification at the low ng/ml level using the closely related mepenzolate bromide as internal standard.

Abuse of glycopyrrolate has caused great concern to horseracing authorities worldwide, with several positive cases having been detected. Administration of the drug usually takes place by intravenous or intramuscular routes at low levels (approximately 4 µg per kg). Excretion in urine is rapid, taking place in less than eight hours, and predominantly in the form of the parent drug. Although ELISA kits are commercially available for this purpose, and provide an effective preliminary screening procedure for drug control, the kits are insufficiently selective for drug detection in sport, where it is mandatory to identify the drug present in the body fluid under test.

Mass spectrometry, however, offers high selectivity, and when coupled with the appropriate ionization method for the sample under scrutiny, also provides high sensitivity. Initial mass spectrometric methods developed for glycopyrrolate involved base hydrolysis of the drug, followed by methylation of the cyclopentylmandelic acid thus produced, and subsequent GC/MS detection of this methyl ester. The various developed methodologies based on this approach have been used to confirm the administration to horses of glycopyrrolate in drug control programmes in equine sports.

Detection of the drug at the Horseracing Forensic Laboratory is currently made using an on-line LC/MS method. However, with the advent of electrospray ionization (ES) mass spectrometry has come the opportunity for the development of an on-line LC/MS method capable of identifying the intact parent drug, and thus the ability to bypass laborious and time-consuming derivatization procedures. Renowned in the biopolymer field, electrospray ionization's reliability and capacity to analyse thermally labile lower molecular weight compounds has ensured its increasing application in the detection and identification of drugs and their metabolites.

*Copies from IAS, Queens Avenue, Macclesfield, Cheshire SK10 2BN, UK. Tel.: 01625 434343; fax: 01625 434335.*

### Flexible MIS and industrial control

Bristol Babcock has announced the formation of Pronexus, its new Software Technologies Division. Pronexus aims to provide industry with the most open, flexible and scaleable business solutions for Management Information Systems (MIS) and industrial control applications. Pronexus will be dedicated to the development of 'next generation' software products, based on the concept of open systems and connectivity and incorporating leading edge software technologies such as object-oriented relational databases, global standards compliance, hardware and software platform independence, database access through standards (for example, SQL and ODBC) and advanced X-Windows operator graphics.

*Details from Pronexus, Worcester Road, Stourport-on-Severn, Worcestershire DY13 9AT, UK. Tel.: 01299 824119; fax. 01299 824118.*

### Portable pH meter

Radiometer's PHM201 Portable pH Meter allows laboratory standard measurements to be taken in the field. The PHM201 automatically recognizes the buffers used for calibration—users have a choice of IUPAC buffers, technical buffers meeting DIN 19267 or buffers pH 4, 7 and 10. There is then the option of single or dual point calibration. The last calibration is also automatically stored to enable electrode performance comparison.

During actual reading, the AUTOREAD function locks the meter onto the signal as soon as it is stable. Measurements can also be real live with the aid of a stability indicator. The measurement range runs from $-9$ to $+23$ pH units (millivolt measurement optional) with a resolution of 0·01 and accuracy of $\pm 0{\cdot}01$ after calibration. There is a continuous range of information available via the display including prompts for the calibration parameters and when to change buffers. The PHM201 operates off battery or mains.y

*For more information contact Ed Lemon at Radiometer Ltd, The Manor, Manor Royal, Crawley, West Sussex RH10 2PY. Tel.: 01293 517599; fax.: 01293 531597.*

### Mercury Measurement at PITTCON '95

At the 1995 Pittsburgh Conference in New Orleans, P.S. Analytical introduced several new products to extend their atomic fluorescence range. Mercury continues to be a major focus of PSA's customers and the new mercury speciation system based on gas chromatography, coupled to atomic fluorescence (GC AFS) is the world's first fully configured instrument to analyse organo-mercury compounds. The system allows rapid analysis of soils, sediments and water samples to ascertain their organo-mercury profile. The methods provide sample extractions which avoid masking any mercury compound present such as diethyl mercury. The system provides detection levels which are better than 1 pg for diethyl mercury.

As a further extension of its product range, P.S. Analytical has introduced two new instruments for on-line mercury analysis. The PSA 10.223 Liquid On-Line System meets clients' needs in the chlor-alkali, chemical or effluent industries. It uses the wide linear dynamic range of the Merlin Detector to analyse such difficult samples on a repeat cycle basis. Systems have already been installed in Europe to monitor effluents 24 hours a day. PSA has also introduced a new Sir Galahad on-line gaseous monitor.

*Further information from Professor Peter Stockwell, P.S. Analytical, Arthur House, Unit 2.03 Crayfields Industrial Park, Main Road, St Pauls Cray, Orpington, Kent BR5 3HP, UK. Tel.: 01689 891211; fax: 01689 896009.*

### Derivative spectroscopy

The theory of derivative spectroscopy is described in a new application note from Hewlett-Packard. The note reviews the fundamental mathematical relationships between analyte concentration and measured absorbance that are used to calculate first and higher-order derivatives. It describes the optical, electronic and mathematical methods of obtaining derivative spectra, and outlines the advantages of the mathematical techniques.

Computer-generated examples are used to illustrate the features and applications of derivative spectroscopy. These examples show how resolution can be increased, how background absorbance and scatter can be eliminated, and how broad absorbing bands and background matrices can be suppressed.

*The application note is available from PMG Logistics, Attn. Petra Butter, P.O. Box 533, 2130 AM Hoofddorp, The Netherlands.*

